# Association between insulin receptor substrate 1 gene polymorphism rs1801278 and gestational diabetes mellitus: an updated meta- analysis

**DOI:** 10.1186/s13098-024-01289-w

**Published:** 2024-03-06

**Authors:** Lili Shen, Junli Liu, Xiaolei Zhao, Aiqin Wang, Xiaomei Hu

**Affiliations:** Department of Obstetrics and Gynecology, Heping Hospital Affiliated to Changzhi Medical College, 110 South Yan’an Road, 046000 Changzhi, Shanxi Province China

**Keywords:** IRS1, Polymorphism, T2DM, Meta-analysis

## Abstract

**Objectives:**

we performed this meta- analysis to investigate the impact of insulin receptor substrate 1 (IRS1) gene rs1801278 on susceptibility to gestational diabetes mellitus (GDM).

**Methods:**

The pooled odds ratio (OR) and 95% confidence interval (95% CI) were calculated, and *p* value is used to determine statistical significance. Sensitivity analysis was performed under three models (dominant, recessive and allele model), and the pooled ORs and 95%CI were calculated. Funnel plots and Begger’s regression test were employed to test the publication bias.

**Results:**

The meta-analysis included 4777 participants (2116 cases and 2661 controls). The *IRS1* rs1801278 (C/T) were not significant associated with GDM risk under the dominant and allele models, OR (95%CI) = 1.22 (0.88–1.70) and 1.24 (0.91–1.68), respectively (both *p* values were more than 0.05). But we also found the *IRS1* rs1801278 (C/T) were significant associated with GDM risk under the recessive model, OR (95%CI) = 0.37 (0.16–0.86), *p* = 0.030. Our results showed that none of the studies affected the quality of the pooled OR. We also found no significant publication bias existed in this meta study for three genetic models, *P*_TT + CT *vs.* CC_ = 0.445; *P*_CC+CT *vs.* TT_= 0.095; *P*_C *vs.* T_ = 0.697.

**Conclusion:**

this meta-analysis indicated that *IRS1* rs1801278 (C/T) was associated with the GDM risk under the recessive model but was not associated with the GDM risk under dominant and allele models.

## Introduction

Gestational diabetes (GDM) refers to abnormal glucose metabolism of different degrees that occurs or is first found during pregnancy, which is characterized by a potential defect in the response of pancreatic beta cells to insulin during pregnancy, which can have adverse effects on perinatal and maternal postpartum outcomes [1, 2]. In addition to these short-term risks, the risk of type 2 diabetes (T2DM) in women with a history of GDM during pregnancy will increase by 17–63% 5 to 16 years after delivery [3]. Both GDM and T2DM are characterized by insulin resistance, there are similarities in their pathogenesis [4], and there is a common genetic background between GDM and T2DM [5].

Insulin receptor substrate 1 (IRS1) gene, which located at chromosome 2q36, is a member of the IRS protein family. It also encodes IRS-1 protein, which plays an important role in signal transmission between insulin receptor and Phosphoinositide 3-kinase (PI3K) [6]. Previous studies have also shown that dysregulation of IRS-1 expression and function can affect the insulin signaling pathway, leading to the occurrence of IR and DM [7, 8]. Previously, several studies have suggested a significant association between IRS-1 gene single nucleotide polymorphisms (SNPs) and T2DM risk [9, 10]. Previous study [11] have confirmed that the substitution of glycine arginine in Gly972Arg (rs1801278) of IRS-1 gene has a statistical correlation with the high incidence rate of GDM. Several studies [12–16] were performed to investigate the relationship between rs1801278 and GDM susceptibility, however these studies concluded inconsistent results. Although, three meta- studies [17–19] have focused on this topic, and the fore- mentioned meta- analysis indicated that rs1801278 was associated with higher GDM risk, but in recent years, there have been new case-control studies [20–22] have been performed, and these new studies concluded inconsistent result with previous meta- analysis. Therefore, we performed this updated meta- analysis to investigate the relationship between IRS-1 gene (rs1801278) and susceptibility to GDM.

## Methods

### Publication search

We systematically searched articles published between 2004 and 2023 on databases of PubMed, Cochrane Library, Web of Science, Embase and China National Knowledge Infrastructure. The following terms were used: (“Gly972Arg” OR “Insulin receptor substrate 1” OR “Insulin receptor substrate” OR “rs1801278”) AND (“pregnancy-induced diabetes” OR “gestational diabetes mellitus” OR “gestational diabetes” OR “pregnancy-induced diabetes mellitus” OR “GDM”). Two staff members independently searched for articles and screened and extracted relevant information from their content.

### Selection criteria

All included studies need to meet the following requirements: (1) evaluation the association between IRS-1 gene rs1801278 and susceptibility to GDM. (2) the studies included should be case-control studies and the full text could be obtained; (3) genotype frequencies for IRS-1 gene rs1801278 could be obtained. The rules for excluding research are as follows: (1) duplication of previous publications; (2) the study was not case- control study; (3) we could not obtain the genotype frequencies for IRS-1 gene rs1801278. PRISMA statement as the preferred reporting rule for this meta-analysis [23].

### Data extraction

The process of literature review and data extraction will be independently conducted by two researchers based on literature selection criteria simultaneously. Information was extracted from these articles, including author, publication year, country, frequencies of *IRS1* rs1801278 genotype and alleles in case and control group. Only articles with the largest sample amount, and the same data appears in multiple publications. To ensure the accuracy and objectivity of data extraction, the third researcher will verify the extracted data with the literature one by one. Any inconsistencies in the data will be publicly decided by the third researcher and the first two researchers together.

### Study quality assessment

The two researchers conducted an independent quality assessment of each qualified article based on the NOS quantity table (NOS), which applied to the quality assessment of observation research. The third appraiser solves the different results of the two appraisers. The evaluation scores mainly include the following aspects: (1) Cases and control options (4 points); (2) the mass of confusion factor in cases and comparison with China correction (2 points); (3) exposure determination (3 points). The total score ranges from 0 to 9, and the score is higher than 6 for high quality.

### Statistical analysis

The OR and 95% CI were calculated to estimate the relevance of *IRS1* rs1801278 (C/T) gene and GDM risk. The Chi-square based Q-test and I-squared test were performed to analyze heterogeneous (*P* < recommended heterogeneity) [24, 25]. When there is no heterogeneity, estimate the combined OR using fixed effects models (Mantel Haenszel) or random effects models (DerSimonian and Laird) [26, 27]. Chi-square test was performed to examine Hardy–Weinberg equilibrium (HWE). Sensitivity analysis was performed for three models (dominant, recessive and allele model) to estimate the influence of the pooled ORs. The publication bias was tested by Funnel plot and Begg’s linear regression [28, 29], *p* < 0.05 was considered existing publication bias. Stata 15.0 was used for all analysis in this study. Two- sides *p*-values less than 0.05 were considered significant.

## Results

### Study characteristics

**Fig. 1:**
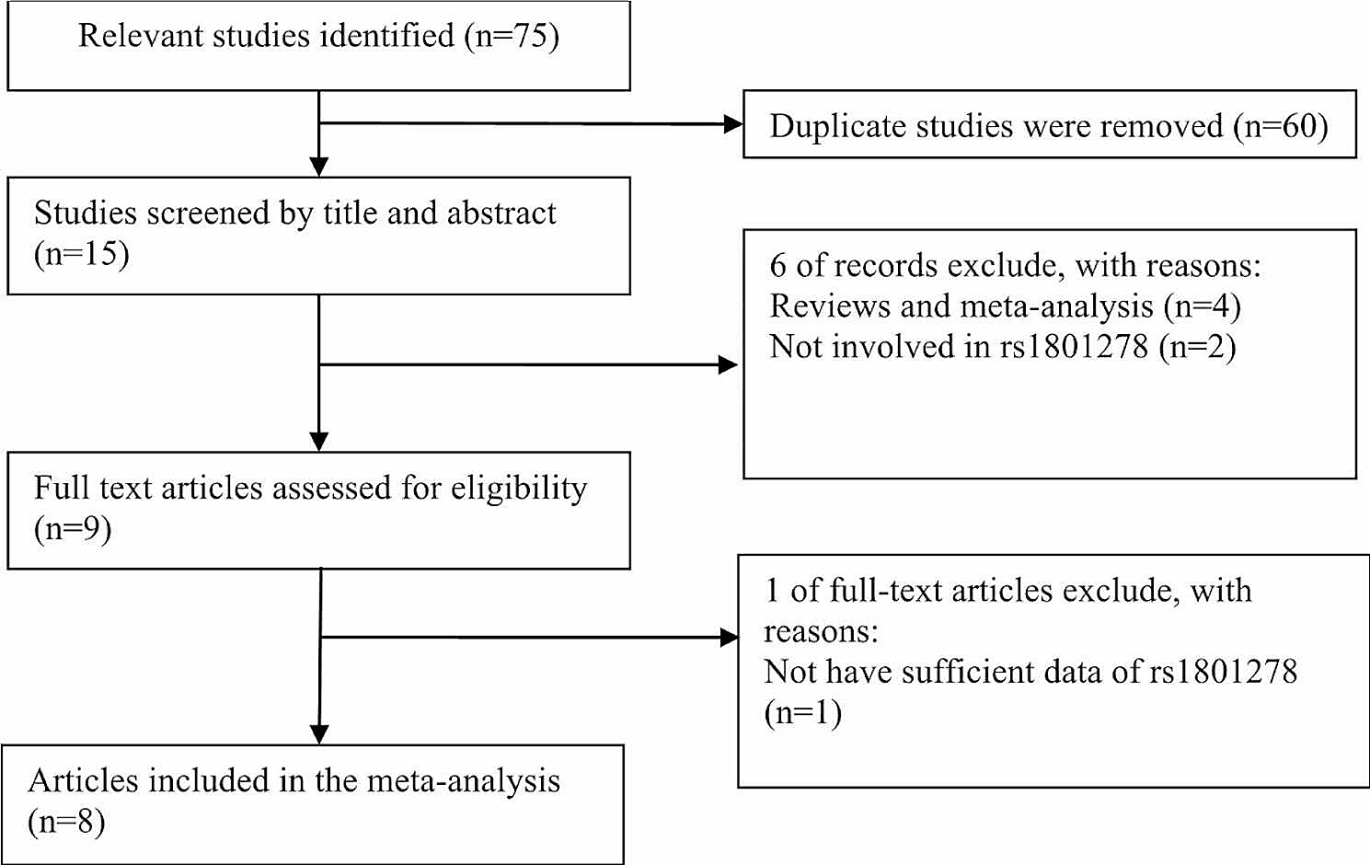
The flow sheet of identification of eligible studies

Figure 1 shows the process of literature search and inclusion. There were 75 publications searched in several electronic databases. After carefully reading the articles, a total of 67 studies were excluded, including 60 duplicate literatures, four meta- studies, two studies not involved in rs1801278 and one study have no sufficient data of rs1801278. Finally, a total of 8 articles were included in current meta-analysis. In these 8 case-control studies, 2 were conducted for Asian populations, 6 were Caucasian. The distribution of genotype for one study did not meet HWE balance (*P* < 0.05), but the distribution of genotype in controls of this study meet the HWE balance (*P* > 0.05). Table 1 shows the main characteristics and HWE test results of all included studies.

**Table 1:** Information for studies included in this meta-analysis

Author	Year	Country	Definition for GDM	Sample size			Case						Control					NOS score	HWE
				Case	Control		CC	CT	TT	C	T		CC	CT	TT	C	T		
Alharbi, et al. ^1^	2014	Saudi	OGTT confirmed	200	300		189	10	1	388	12		295	5	0	595	5	6	0.022
Pappa, et al. ^1^	2011	Greece	Fourth IWCGDM criteria	148	107		58	73	17	189	107		60	40	7	160	54	8	0.682
Tok, et al. ^1^	2006	Turkey	NDDG criteria	62	100		53	9	0	115	9		89	11	0	189	11	6	0.402
Fallucca, et al. ^1^	2006	Italy	OGTT confirmed	309	277		271	34	4	576	42		255	22	0	532	22	8	0.071
Shaat, et al. ^1^	2005	Sweden	EASD-DPSG criteria	588	1189		534	49	4	1117	57		1078	111	0	2267	111	8	0.986
Popova, et al. ^2^	2021	Russians	IADPSG criteria	319	318		292	27	0	611	27		284	34	0	602	34	6	0.204
Wu, et al. ^2^	2021	Chinese	IADPSG criteria	213	191		205	8	0	418	8		184	7	0	375	7	6	0.704
Popova, et al. ^2^	2017	Russians	IADPSG criteria	278	179		257	21	0	535	21		160	19	0	339	19	6	0.328

Note: 1: those articles were included in previous meta- analysis; 2: those articles were not included in previous meta- analysis. OGTT: the oral glucose tolerance test; IADPSG: the International Association of Diabetes in Pregnancy Study Groups; EASD-DPSG: the European Association for the Study of Diabetes-Diabetic Pregnancy Study Group; IWCGDM: International Workshop-Conferences on Gestational Diabetes Mellitus; NDDG: National Diabetes Data Group criteria

### Meta-analysis results

**Fig. 2:**
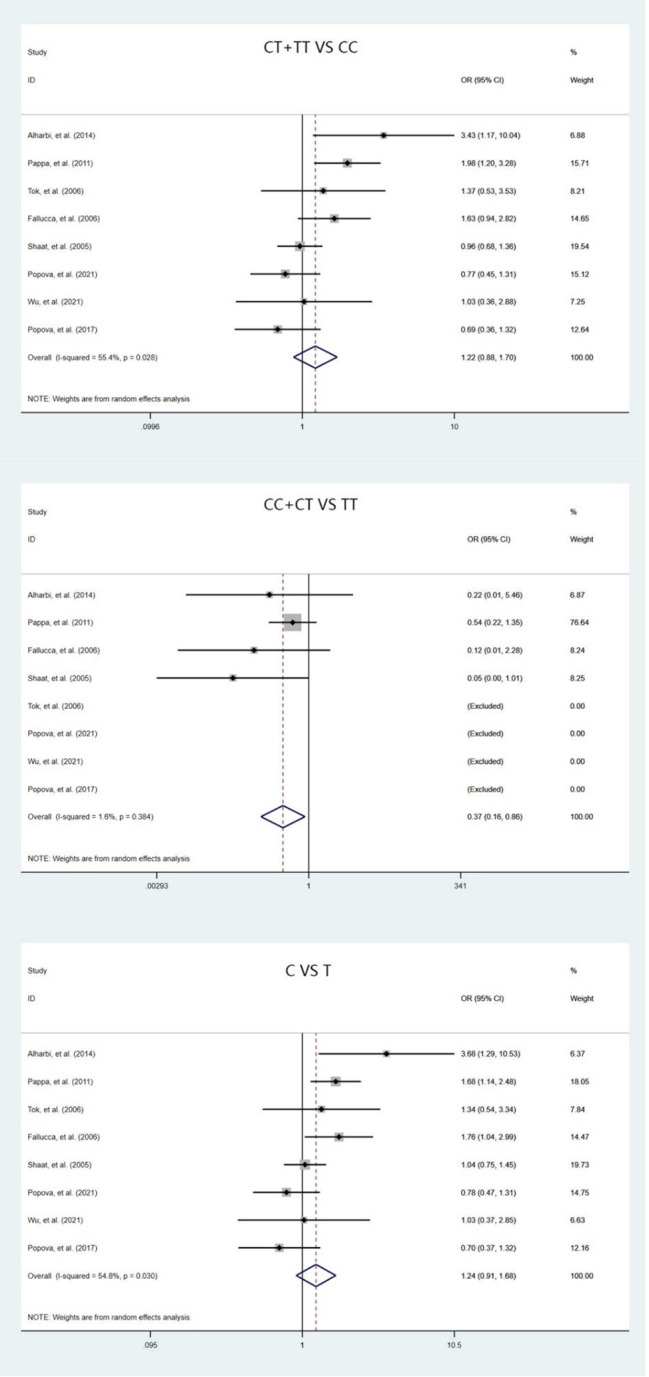
Forest plots of the *IRS1 rs1801278 (C/T)* polymorphism under three genetic models (dominant, recessive and allele model)

The meta-analysis included 4777 participants (2116 cases and 2661 controls). The *IRS1* rs1801278 (C/T) were not significant associated with GDM risk under the dominant and allele models, OR (95%CI) = 1.22 (0.88–1.70) and 1.24 (0.91–1.68), respectively (both *p* values were more than 0.05). But we also found the *IRS1* rs1801278 (C/T) were significant associated with GDM risk under the recessive models, OR (95%CI) = 0.37 (0.16–0.86), *p* = 0.030 (Fig. 2).

### Sensitivity analysis

**Fig. 3:**
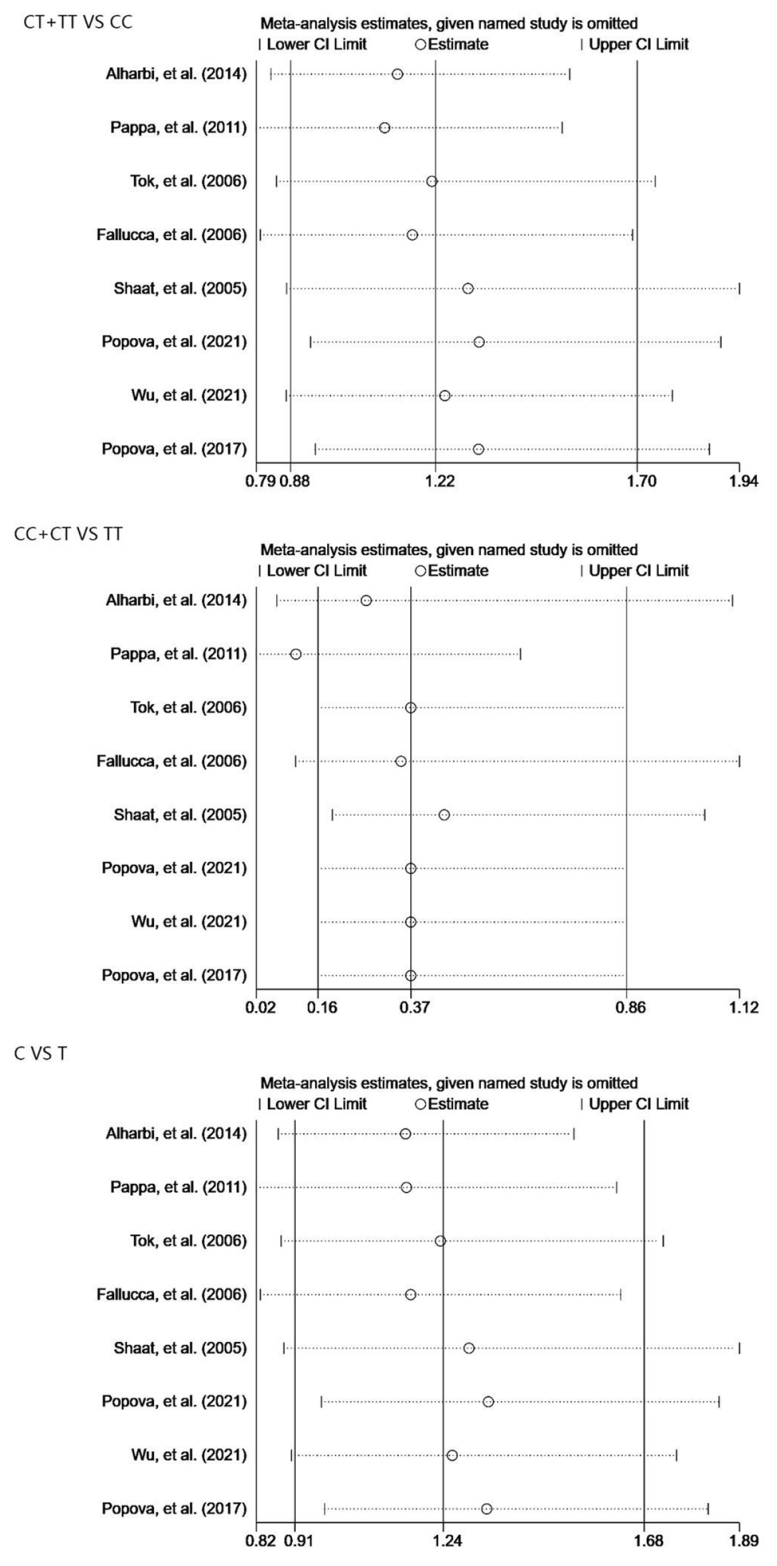
Sensitivity analysis examining the association between the *IRS1 rs1801278 (C/T)* and risk of gestational diabetes mellitus under three genetic models (dominant, recessive and allele model)

Sensitivity analyzes were performed by sequentially removing each eligible study one at a time to assess the effect of each study on the pooled OR. Our results showed that none of the studies affected the quality of the pooled OR (Fig. 3).

### Publication bias

**Fig. 4:**
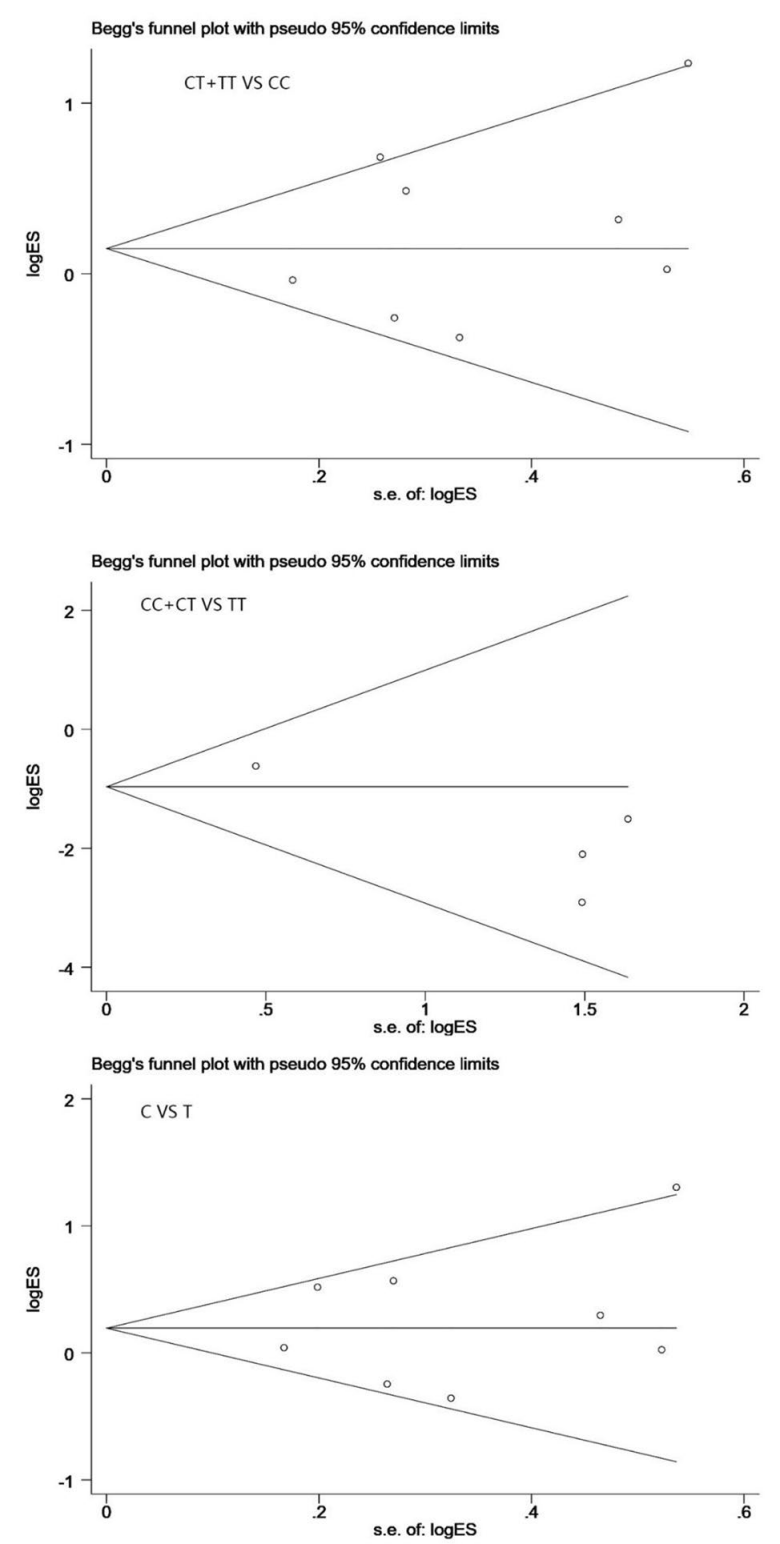
Begg’s funnel plot for publication bias analysis under three genetic models (dominant, recessive and allele model)

Publication bias was evaluated using the Begg’s funnel plot. We found no significant publication bias existed in this meta study for three genetic models, *P*_TT + CT *vs.* CC_ = 0.445; *P*_CC+CT *vs.* TT_= 0.095; *P*_C *vs.* T_ = 0.697 (Fig. 4).

## Discussion

In current meta- study, we found that the *IRS1* rs1801278 (C/T) were not significant associated with GDM risk under the dominant and allele models, but the *IRS1* rs1801278 (C/T) were significant associated with GDM risk under the recessive models. Several studies [12–16] were performed to investigate the relationship between rs1801278 and GDM susceptibility, however these studies concluded inconsistent results. A case- control [12] study performed in Saudi women suggested that the IRS-1 rs1801278 variant may associated with increased risk of GDM under the dominant model. A study [13] for Greek population suggests that the risk of developing GDM is higher among female carriers of the minor allele within IRS1 gene rs1801278. But Shaat et al. [14] concluded inconsistent results, they found a negative result on relationship between IRS1 gene rs1801278 and GDM risk. Although, three meta- studies [17–19] have focused on this topic, and the three meta- analyses indicated that rs1801278 was associated with higher GDM risk, but in recent years, there have been new case-control studies [20–22] have been performed, and these new studies concluded inconsistent result with previous meta- analysis. Wu et al. [19] conducted a meta study suggested that GDM was associated with rs1801278(IRS1), but this relationship was not significant in Asian populations. In addition, the IRS1- rs1801278 was significantly affected by OGTT protocol and genotyping methods. Zhang et al. [17] indicated that the minor alleles of IRS1- rs1801278 (Gly972Arg) were significantly associated with a higher risk of GDM, and another meta- study concluded similar results. From then on, additional case- control studies were performed. Popova et al. [20] suggested that the distribution of genotype frequencies was not significant different between cases and controls. In 2021, Popova et al. [21] conducted another case- control study in Russian women and they obtained similar results. Wu et al. [22] performed a case- control study in Chinese females and found that there was no statistically significant difference in genotype frequency between the case and the control group.

Previous research [30, 31] suggests that IRS1 is a substrate of insulin receptor tyrosine kinase and plays a crucial role in insulin signaling pathways. IRS1 protein can be expressed in many insulins sensitive tissues, and its tyrosine phosphorylation can trigger activation of phosphatidylinositol 3-kinase (PI3K) and translocation of glucose transporter [32]. Evidence [14] have shown that the IRS1 G972R polymorphism reduces insulin content in isolated human islets and impairs insulin secretion function.

Several limitations existed in this meta-analysis. Firstly, we could not analyze the data grouped by ethnic, because just two studies were conducted in Asian. Secondly, the pathogenesis of GDM is very complex, including genetic factors, environmental factors and the synergistic effects. Therefore, this study only analyzes the association between a single SNP and GDM, without involving gene- environmental synergistic effects.

In conclusion, this meta-analysis indicated that *IRS1* rs1801278 (C/T) was associated with the GDM risk under the recessive model but was not associated with the GDM risk under dominant and allele models.

## Data Availability

No datasets were generated or analysed during the current study.

## Abbreviations

GDM: Gestational diabetes mellitus
HWE: Hardy–Weinberg equilibrium
IRS1: Insulin receptor substrate 1
PRISMA: Preferred Reporting Project
SNP: Single nucleotide polymorphism
T2DM: Type 2 diabetes
